# Combination of Biochar and Functional Bacteria Drives the Ecological Improvement of Saline–Alkali Soil

**DOI:** 10.3390/plants12020284

**Published:** 2023-01-07

**Authors:** Shuang Liang, Sheng-Nan Wang, Lu-Lu Zhou, Shuo Sun, Jian Zhang, Lin-Lan Zhuang

**Affiliations:** 1 Shandong Key Laboratory of Water Pollution Control and Resource Reuse, School of Environmental Science & Engineering, Shandong University, Qingdao 266237, China; 2 Baiyangdian Basin Eco-environmental Support Center, Shijiazhuang 050000, China; 3 College of Safety and Environmental Engineering, Shandong University of Science and Technology, Qingdao 266590, China

**Keywords:** saline–alkali soil, plant-growth-promoting rhizobacteria, biochar, soil fertility, soil microbiome, plant growth

## Abstract

The addition of functional bacteria (FB) is low-cost and is widely applied in saline–alkali soil remediation, which may gradually become ineffective due to inter-specific competition with indigenous bacteria. To improve the adaptability of FB, the target FB strains were isolated from local saline–alkali soil, and the combined effects of FB and biochar were explored. The results showed that FB isolated from local soil showed better growth than the purchased strains under high saline–alkali conditions. However, the indigenous community still weakened the function of added FB. Biochar addition provided a specific niche and increased the relative abundance of FB, especially for *Proteobacteria* and *Bacteroidota*. As a result, the co-addition of 10% biochar and FB significantly increased the soil available phosphorus (AP) by 74.85% and available nitrogen (AN) by 114.53%. *Zea Mays’s* growth (in terms of height) was enhanced by 87.92% due to the decreased salinity stress and extra nutrients provided.

## 1. Introduction

Worldwide soil salinization is considered to be one of the most significant environmental problems [1]. Saline soil, due to its large amounts of exchangeable sodium, barren soil fertility, and poor microbial activity [2], seriously limits crop productivity. There are about 3.67 × 10^7^ ha of saline soil in China [3], accounting for 5.0% of the available land in the country [4]. It has been estimated that the demand for food will increase by 70% worldwide within 30 years owing to population growth [5], which will increase pressure on the finite amount of cultivated land [6]. Saline–alkali land is an alternative land resource which could be developed for agricultural use to alleviate the food crisis [7]. Various techniques (e.g., physical, chemical, and biological technologies) have been attempted for saline soil remediation [8,9,10]. Among them, bioremediation technology has the advantages of low cost, no secondary pollution, and high sustainability [3].

The soil environment contains diverse microbiomes. The root systems of plants play a crucial role in salt–alkali tolerance and nutrient utilization [11]. Plant-growth-promoting rhizobacteria (PGPR) are a form of functional bacteria (FB) that can promote the utilization of mineral nutrients and subsequent plant growth [12]. Previous studies have demonstrated that PGPR can fix nitrogen [13], solubilize phosphate [14], produce siderophore [15], induce nutrient acquisition, and gain systemic resistance against environmental pressure [16] to accelerate plant growth. As salt-tolerant plant-growth-promoting rhizobacteria (ST-PGPR) isolated from extreme saline–alkaline soils, *Azospirillum, Rhizobium*, *Bacillus*, *Agrobacterium*, and *Paenibacillus* have been shown to greatly improve crop yields under high-saline conditions [17,18,19]. Researchers have continued to study extreme-environment-tolerant FB and explore its tolerant mechanisms for better application in agro-ecosystems [12,19]. It has been widely proved that the inoculation of an FB consortium can reshape the structure of the soil microbial community. However, the FB consortia used in previous experiments were almost purchased from microbiological culture collection centers or isolated from other regions previously. The functions of bacteria isolated from local soil have rarely been reported. The effect of native microbial communities on additional FB is always ignored [20,21,22] and their long-term compatibility needs to be investigated. Although FB are effective in improving soil properties once added, they may gradually decrease due to the rejection of native micro-organisms. FB isolated from local soil are supposed to show high compatibility with local communities, which may demonstrate relatively sustainable performance on soil remediation. However, there are few reports of these studies.

Biochar possesses rich carbon, a large specific surface area, and a highly developed pore structure, and has received extensive attention in the field of environmental remediation [23,24]. Biochar can modify soil through physical processes (increasing cation exchange capacity (CEC)) [25], chemical processes (reducing N leaching and absorbing soluble nutrients) [24,26], and biological processes (increasing microbial abundance and improving microbial communities) [27]. Recently, increasing numbers of studies have demonstrated that biochar application in saline–alkali soil alleviates salinity stress by reducing Na^+^ uptake and promotes plant growth [28,29]. To our knowledge, there have been no studies evaluating the combined effect of indigenous FB and biochar in saline–alkali land remediation.

Considering that biochar can provide attachment sites and a favorable environment for FB growth, biochar and FB isolated from local saline–alkali soil were combined to drive the ecological remediation of saline–alkali soil in this study. Therefore, this study investigated: (1) the advantages of FB isolated from local soils; (2) the interaction between native micro-organisms and FB; (3) the function of biochar on FB colonization; and (4) the combined effects of FB and biochar on soil physicochemical properties, microbial community structure, and plant metabolic processes.

## 2. Results

### 2.1. Isolation, Identification and Tolerance Test of FB

Two pure strains named FN2 and DP3 were isolated through Ashby and PKO media, respectively. After culturing on LB medium at 30 °C for 48 h, both strains formed colonies with round and smooth edges (Figure S1a,b). Using electron microscope detection, the average size of FN2 was estimated to be (1.3~1.5) μm × (0.6~0.7) μm, and the average size of DP3 was (1.5~3.0) μm × (0.6~1.0) μm (Figure S1c,d). Both FN2 and DP3 were aerobic Gram-negative rod-shaped non-spore bacteria. Physiological and biochemical properties (Tables S1 and S2) showed that FN2 and DP3 could use a variety of carbon sources, including carbohydrates (such as D-Trehalose, D-Fructose, D-Fucose, D-Mannitol, and L-Rhamnose) and amino acids (such as L-Alanine, L-Aspartic Acid and L-Glutamic Acid), among others, which allowed them to adapt to the barren soil environment. 16S rDNA sequencing revealed that DP3 was *Agrobacterium sp.* and FN2 was *Rhizobium rosettiformans* (Figure S1e,f). Strains DP3 (CGMCC No. 21306) and FN2 (CGMCC No. 21307) were sent for preservation in the China General Microbiological Culture Collection Center (CGMCC).

**Figure 1:**
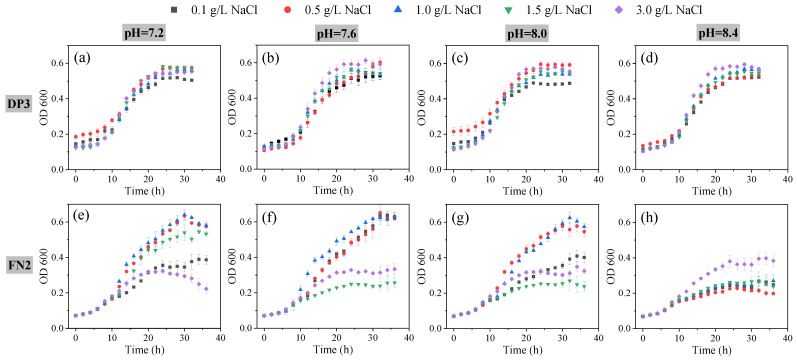
The growth curves of FN2 and DP3 under various salinity and alkalinity stresses. (**a**) DP3 under pH = 7.2; (**b**) DP3 under pH = 7.6; (**c**) DP3 under pH = 8.0; (**d**) DP3 under pH = 8.4; (**e**) FN2 under pH = 7.2; (**f**) FN2 under pH = 7.6; (**g**) FN2 under pH = 8.0 and (**h**) FN2 under pH = 8.4.

Before applying FB to the saline–alkali soil, the growth of the isolated bacteria under different salinity and alkalinity conditions was investigated and is shown in Figure 1. DP3 followed a typical S-shaped growth curve and reached a stationary phase after approximately 24 h of culture (Figure 1a–d). As shown from the aspect of bacterial density at the stationary phase, the growth of DP3 was the worst under 0.1 g/L NaCl. Compared with 0.1 g/L NaCl, 3.0 g/L NaCl significantly increased the DP3 density by 16.68%, 18.20%, and 13.87% (*p* < 0.05) when pH values were 7.6, 8.0, and 8.4, respectively. Meanwhile, DP3 had the highest specific growth rate (0.052 h^−1^) at the salinity of 3.0 g/L and pH of 8.4. That is to say, high salinity and alkalinity had no negative effect on DP3 growth.

Compared with DP3, high salinity and alkalinity had a relatively larger influence on FN2 growth (Figure 1e–h). FN2 entered the stationary phase in advance under too low or too high salinity (≤0.1 g/L or ≥1.5 g/L), while moderate salinity (0.5 g/L NaCl and 1.0 g/L NaCl) stimulated the growth of FN2, and FN2 densities of the stationary phase under moderate salinity were more than twice as much as those under extreme salinity. Moreover, FN2′s growth was greatly inhibited under a pH of 8.4. This indicated that the growth of FN2 was not inhibited when the pH ranged from 7.2 to 8.0 and salinity from 0.1 g/L NaCl to 1.0 g/L NaCl. FN2 showed a relatively narrow range of saline–alkali tolerance compared with DP3.

### 2.2. Effects of Biochar and FB on the Ecology of Saline–Alkali Soil

#### 2.2.1. Soil Physicochemical Properties

Based on the high saline–alkali tolerance of the two isolated FB, the mixed FB (mentioned in Section 4.2) were added to the saline–alkali soil for remediation. The changes in soil properties after FB application are listed in Table 1. Adding FB to sterilized soil (SSPB) significantly enhanced TN by 29.41% and AN by 31.36% (*p* < 0.05), while it had little improvement in unsterilized soil (SPB). The effects of the combined addition of FB and biochar (with dosages of 1%, 5%, and 10%) were explored comprehensively. The results showed that the combined enhancement by biochar and FB on saline–alkali soil fertility was superior to single FB addition (Table 1). Compared with SP, the co-addition of FB and 1% biochar (SPBC1) significantly enhanced the soil TN and AN by 41.18% and 61.82%, respectively (*p* < 0.05). Furthermore, TP, TN, SOM, AP, and AN contents were all boosted with the increase in biochar dosage, resulting in an enhancement of 26.56%, 76.47%, 111.73%, 74.85%, and 114.53% in group SPBC10, respectively (*p* < 0.05). The co-addition of biochar and FB also had a significant influence on the soil’s physical properties and soluble salt. Biochar addition significantly increased the soil porosity by 6.46% in SPBC5 and 20.55% in SPBC10, and the soil moisture content increased by 16.85% in SPBC5 and 13.35% in SPBC10 (*p* < 0.05). A total of 10% biochar and FB addition significantly decreased the salt content of soil by 17.15% (*p* < 0.05).

#### 2.2.2. Soil Microbial Community Composition

Diversity is a comprehensive index of species richness and evenness. Operational Taxonomic Units (OTUs) number, Chao1, and Abundance-based Coverage Estimator (ACE) indices were used to estimate species richness, while Shannon and Simpson indices indicated species diversity [7,30]. The alpha diversity of microbial community composition (Table S3) showed that soil without any treatment (S) had the highest OTUs number, Chao1, ACE, Shannon, and Simpson indices, while the addition of plants or FB decreased the OTUs number in different degrees.

**Figure 2:**
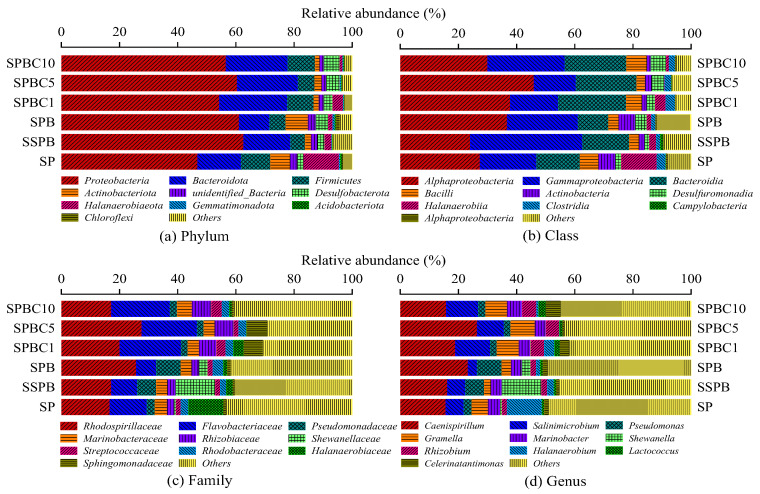
Top 10 microbial community composition distribution at (**a**) phylum, (**b**) class, (**c**) family, and (**d**) genus level. Notes: “S” represents “unsterilized soil”, “SS” represents “sterilized soil”, “P” represents “plant”, “B” represents “functional bacteria”, “C” represents “biochar”, and the number following the abbreviation represents the proportion of biochar mass to soil mass.

Further analysis was made on the microbial community composition at different taxonomic levels (Figure 2). At the phylum level, the dominant bacterium was *Proteobacteria* (46.82~62.63%), followed by *Bacteroidota* (10.48~23.39%), *Firmicutes* (5.01~10.10%), *Actinobacteriota* (1.50~7.60%), and others (Figure 2a), which were a typical saline–alkaline soil microbiome [31]. FB inoculation (SPB) further consolidated the dominant position of *Proteobacteria* and increased its relative abundance by 14.28%. Biochar and FB addition could affect the quantity of various bacteria species and affect microbial community diversity. *Bacteroidota* has been reported to be enriched in soil with high carbon [32]. The relative abundances of *Bacteroidota* in biochar- and FB-added soils (SPBC1, SPBC5, and SPBC10) were the highest among all groups (Figure 2a), which corresponded to the higher SOM content than that in SP (*p* < 0.05) (Table 1). The co-addition of FB and biochar increased the relative abundance of *Proteobacteria* and *Bacteroidota* by 7.50~13.65% and 5.90~8.41%, while it reduced the relative abundance of *Firmicutes* and *Actinobacteriota* by 0.65~4.38% and 4.29~5.19% compared with SP. At the class level, *Alphaproteobacteria*, *Gammaproteobacteria* (classified as *Proteobacteria)*, combined with *Bacteroidia* and *Actinobacteria*, formed the dominant bacteria at the class level in saline–alkali soil as Zhao et al. reported (Figure 2b) [33].

The top 10 microbial community compositions at the genus level are shown in Figure 2d. The dominant bacteria, such as *Caenispirillum*, *Salinimicrobium*, *Pseudomonas*, *Gramella*, and *Marinobacter*, are typically halophilic micro-organisms [31,34,35]. Microbial community composition indicated that salinity and alkalinity played an important role in the construction of the soil microbial community, which coincided with Zhao’s study [33]. *Caenispirillum* belongs to *Rhodospirillaceaei,* and *Proteobacteria*, which was reported to be isolated from a salt desert, could tolerate an extremely high-salinity (*w*/*v*, 0~12%) and -alkalinity (pH, 7~10) environment [36]. The leanness-tolerant and salinity-tolerant ability made *Caenispirillum* occupy the dominant position though it cannot increase the N and P contents of soil [37]. Compared with SP, the combined effect of biochar and FB expanded the dominant advantage of *Caenispirillum*, *Salinimicrobium*, *Gramella*, and *Rhizobium*, while reducing the quantity of *Halanaerobium*. The relative abundance of the added FB at the genus level is also summarized in Table 2. Groups with biochar addition (i.e., SPBC1, SPBC5, and SPBC10) were compared with groups without biochar (i.e., S, SP, and SPB). The results showed that *Bacillus*, *Azospirillum*, and *Rhizobium* were significantly increased by biochar addition (*p* < 0.05).

#### 2.2.3. Plant Growth and Physiological Indices

**Figure 3:**
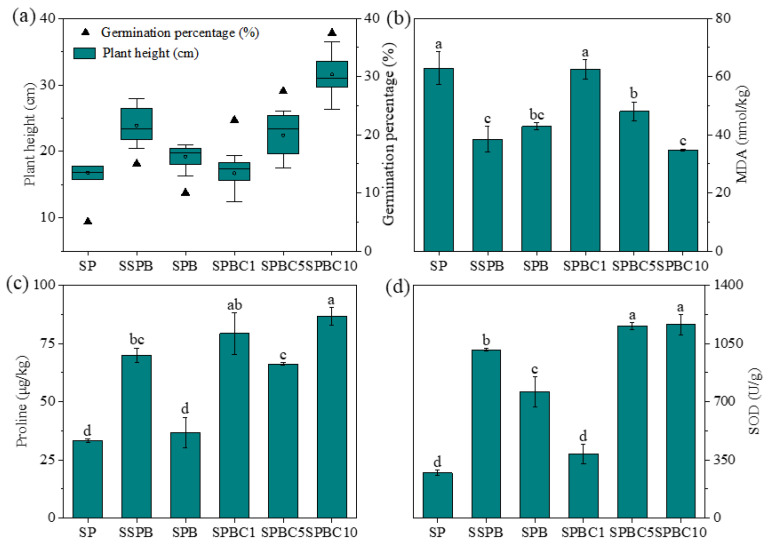
Maize growth and physiological indices in different treatments. (**a**) Plant height and germination percentage; (**b**) MDA content; (**c**) Proline content; (**d**) SOD content. Notes: Different letters indicate significant differences between different treatments (*p* < 0.05). “S” represents “unsterilized soil”, “SS” represents “sterilized soil”, “P” represents “plant”, “B” represents “functional bacteria”, “C” represents “biochar”, and the number following the abbreviation represents the proportion of biochar mass to soil mass.

Based on the previous results, it appears that the combined addition of biochar and FB could enhance soil nutrients and optimize the microbial community structure. The effects of FB and biochar on maize growth and metabolic process were further explored (Figure 3). Due to the high salinity and the poor nutrition of saline–alkali soil, the germination rate of maize in the SP group was only 5% and the average plant height was only 16.80 ± 1.41 cm after 21 days of cultivation. Plant growth in sterilized soil with FB inoculation (SSPB) was significantly promoted, with plant height increasing by 42.26% (*p* < 0.05) compared with SP, while the promotion effect was not significant in non-sterilized soil with FB inoculation (SPB) (*p* > 0.05). Within the groups of combined application of biochar and FB, plant heights and germination rates all improved with the increase in biochar content. The combination of 10% biochar and FB achieved the optimal effect, and significantly enhanced plant height and germination rates in saline–alkali soil by 87.92% and 32.50%, respectively.

The metabolic process of plants was determined to further investigate how biochar and FB co-addition influence the saline–alkali resistance ability of maize. Salt stress induces the accumulation of reactive oxygen species (ROS). When ROS reach a certain threshold, lipid peroxidation occurs [38]. MDA content reflects the degree of lipid peroxidation, and enzymatic antioxidant systems, such as SOD, could neutralize the accumulated ROS [38]. Furthermore, osmoregulatory substances such as proline could improve the osmoregulatory ability of plant cells and increase plant resistance against stress [38]. Figure 3b shows that SP had the highest MDA content (62.95 nmol/kg), and FB addition significantly reduced MDA content by 38.90% in SSPB and 31.93% in SPB (*p* < 0.05). Contrary to the trend of MDA, proline and SOD content were lowest in SP (Figure 3c, d). SSPB significantly increased leaf proline and SOD content by 109.60% and 273.69%, compared with SP. MDA gradually decreased with the increase in biochar. SPBC10 significantly decreased MDA by 44.67% and increased leaf proline and SOD content by 160.29% and 328.18% (*p* < 0.05).

**Figure 4:**
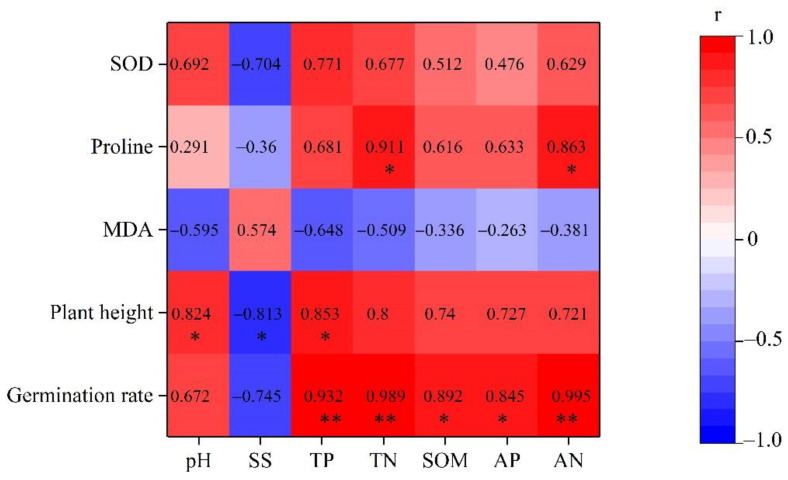
Heat map of the Pearson correlation analysis between maize physiological indices and soil physiochemical properties. *, *p* < 0.05, **, *p* < 0.01. SS means soluble salts.

Correlations between maize growth and soil quality are analyzed in Figure 4. The plant germination rate was positively correlated with soil nutrient level (r > 0.8, *p* < 0.05). Plant height was positively correlated with TP (r = 0.853, *p* < 0.05), and proline content in leaves was positively correlated with nitrogen content in soil (r > 0.8, *p* < 0.05).

## 3. Discussion

### 3.1. The Superiority of Isolated Functional Bacteria

The isolated bacteria DP3 grew well under high salinity (up to 3.0 g/L). However, the growth of the purchased FB strain *Bacillus megaterium* was seriously inhibited under saline–alkali conditions (salinity of 3.0 g/L and pH of 8.4, Figure S2a). The isolated bacteria FN2 also showed better tolerance of the saline–alkali condition than the purchased *Azospirillum brasilense* (Figure S2b). In Dongying city, the moderate saline–alkali soil (salinity of 2.0–4.0 g/kg and pH between 8.09–8.35) accounted for more than half of the land area [39]. The isolated bacterial strains survived in this kind of soil after long-term natural selection, which could adapt to high saline–alkali conditions better than the other purchased strains [19]. Due to their high growth potential, the isolated bacteria from local soil were assumed to have superiority in nutrient supplements and be more suitable for saline–alkali soil remediation than the purchased strains. This viewpoint was also supported by other papers [19,40].

### 3.2. The Disadvantage of Single Application of Bacterial Remediation

The changes in fertility from FB addition in sterilized/unsterilized soils indicate that FB had the potential to improve nutrient content in saline–alkali soil. However, the presence of various indigenous micro-organisms competed with FB for survival resources, and the growth and function of FB could be inhibited [40]. The relative abundance of added FB in sterilized soil was higher than that in unsterilized soil (Table 2), which proved the above conjecture. If FB was lost from the soil ecosystem, it would not supply nutrients to the soil and plants sustainably; this is the disadvantage of a single application of bacterial remediation. Considering the porosity of biochar, it may serve as a shelter for FB and enhance its function, which was further investigated in our study.

### 3.3. Function of FB and Biochar on the Ecology of Saline–Alkali Land 

#### 3.3.1. The Direct Function of Biochar for Saline–Alkali Land Remediation

Soil fertility and porosity gradually increased and salt decreased with the increase in biochar dosage. Biochar with various nutritional elements can serve as a source of available nutrients for soil [41]. The developed pore structure of biochar also enhanced the water-holding capacity of soil indirectly [42,43]. The reasons for the reduced soil salinity may be: (1) the direct dilution effect by partial replacement of biochar [44]; (2) the direct adsorption by biochar [45,46]; (3) the replacement of exchangeable Na^+^ by Mg^2+^ and Ca^2+^ in biochar [44,47,48]; or (4) enhanced eluviation by soil structure changes [49].

#### 3.3.2. The Interaction between Biochar and FB for Saline–Alkali Land Remediation

Though biochar is rich in carbon, phosphorus, and other nutrient elements [24,26], the contents of nitrogen and phosphorus were limited and the release of nutrients was not sustainable. Biochar can play a long-term role in improving soil fertility only when it is combined with colonizing micro-organisms and can support the micro-organisms to produce nutrients sustainably. Our study showed that a 10% biochar addition (SPBC10) increased the relative abundance of FBs. *Bacillus*, *Paenibacillus*, *Azospirillum*, and *Rhizobium* were increased by 1.65, 1.20, 3.30, and 3.19 times, respectively, compared with SP (Table 2). This may be because the porous biochar effectively prevented the loss of FB [30] and provided favorable environmental conditions for FB operation, which further improved the soil’s nutrient content [29] and optimized microbial community diversity [43]. Additionally, it can be seen that biochar created specific niches and had better colony-promoting effects on *Azospirillum* and *Rhizobium*. The combined action of biochar and FB significantly increased the soil’s nitrogen content, and nitrogen-fixing bacteria prolonged the promoting effect (Table 1). The bacteria-regulating effects of biochar were also reported by Cui in chicken manure composting [27], which indicates the high potential of biochar applications.

### 3.4. Combined Function of FB and Biochar on Plant Growth and Physiological Adjustment

The better plant growth of SSPB compared with that of SPB also showed the inhibited function of FB due to indigenous bacteria, while, when combined with biochar, plants showed the best growth. High salinity influenced the nutrient uptake of plants, disturbed plant physiological processes, and restricted plant growth [47]. Plant physiological properties revealed that the harsh salt environment caused serious oxidative damage to plants [19], while the combined addition of FB and biochar alleviated the adverse environmental stress on plants by increasing SOD and proline.

In general, biochar regulated soil porosity, reduced soil salinity [28,29], and provided a specific niche for selected FB adherence. FB increased soil N and P content sustainably with the co-operation of biochar [21]. The combined effect of biochar and FB improved plant growth mainly through adjusting the soil’s physicochemical properties (especially nutrient level) and further influenced plant physiological processes against salinity stress. The combined application of biochar and FB was a vital and effective measure for saline–alkali soil remediation.

### 3.5. Potential Problems and Solutions of the Combined Biochar and FB for Soil Remediation

Though the application of biochar in soil remediation has been widely studied [23,24], and the effect of combined FB and biochar on saline–alkali soil remediation was satisfying in our study, the cost of biochar would be prohibitive for further scaled-up applications. Taking 10% biochar as an example [43], the dosage of biochar would be about 20 t/ha and the cost would be up to USD 20,000/ha. Improving the soil remediation efficiency of a given weight of biochar by surface modification or porosity enlargement may be one solution for reducing the dosage of biochar and the subsequent cost [24]. Besides biochar function, the enhancement of microbial function (e.g., constructing a complex functional bacteria community instead of adding several bacteria strains) would be another method of sustainable and high-efficiency soil remediation.

## 4. Materials and Methods

### 4.1. Chemicals and Culture Medium

Ashby’s medium and Pikovaskaia’s culture medium (PKO) were used to select nitrogen-fixing bacteria and phosphorus-solubilizing bacteria, respectively. Ashby’s medium contains the following chemicals (g/L): KH_2_PO_4_ 0.2, MgSO_4_·7H_2_O 0.2, NaCl 0.2, CaCO_3_ 5.0, mannitol 10.0, CaSO_4_·2H_2_O 0.1, agar 20.0 (pH 7.0). PKO medium contains (g/L): glucose 10, Ca_3_(PO_4_)_2_ 5.0, (NH_4_)_2_SO_4_ 0.5, NaCl 0.2, KCl 0.2, MgSO_4_·7H_2_O 0.3, MnSO_4_·H_2_O 0.03, FeSO_4_·7H_2_O 0.03, yeast extract 5.0, agar 20 (pH 7.0). Luria–Bertani (LB) medium contains (g/L): tryptone 10.0, yeast extract 5.0, NaCl 10.0 (pH 7.0). The above media were sterilized at 121 °C for 30 min before use and all operations were carried out in a clean bench under sterile operating conditions.

### 4.2. Isolation, Identification and Performance of FB

Rhizosphere and non-rhizosphere soil samples were collected from *Suaeda-salsa*-cultivated saline–alkali land (37°53’ N, 118°29’ E) in August 2020, which is located in Dongying city, Shandong province, China. Rhizosphere soil used for screening bacterium strains was collected from the rhizosphere of *Suaeda salsa*, while non-rhizosphere soil used for pot experiments was selected from the topsoil (0–20 cm). Both soil samples were crushed and screened with a 2 mm sieve and thoroughly homogenized. The physicochemical properties of non-rhizosphere soil were as follows: pH 8.65 ± 0.06, soil-soluble salt 3.25 ± 0.18 g/kg, soil organic matter (SOM) 3.19 ± 0.21 g/kg, total phosphorus (TP) 0.61 ± 0.03 g/kg, total nitrogen (TN) 0.16 ± 0.02 g/kg, available phosphorus (AP) 1.47 ± 0.16 g/kg, and available nitrogen (AN) 15.63 ± 0.62 g/kg.

To create a soil suspension, 10 g of rhizospheric soil was added to 90 mL of sterilized deionized water and subsequently oscillated at 160 rpm for 20 min. The diluted soil suspension (10^−4^, 10^−5^, 10^−6^ and 10^−7^) was inoculated in Ashby’s medium or PKO medium with the spreading plate method and incubated at 30 °C for FB isolation. After that, a single colony was selected and purified on Ashby medium or PKO medium for more than 4 times. The isolated strains with the ability to fix nitrogen and dissolve phosphorus were named FN2 and DP3, respectively. Then, the cell morphology, physiological and biochemical characteristics, and taxonomic identifications were detected. The other four functional bacteria with the ability to dissolve phosphorus or fix nitrogen (*Bacillus megaterium* (CGMCC No. 1.0217), *Azospirillum brasilense* (CGMCC No. 1.10379), *Bacillus subtilis* (CGMCC No. 1.1086), and *Paenibacillus mucilaginosus* (CGMCC No. 01075)) were purchased from the China General Microbiological Culture Collection Center (CGMCC). The growth curves of these bacteria under different salinity and alkalinity stresses were determined every two hours until bacteria entered stationary phase. pH gradients were set as 7.2, 7.6, 8.0, and 8.4, and NaCl was added with dosages of 0.1 g/L, 0.5, g/L, 1.0 g/L, 1.5 g/L, and 3.0 g/L, respectively.

### 4.3. Design of Pot Experiments 

A total of six treatments were designed for pot experiments, and the specific experimental design is shown in Table 3.

Sterilized soil was acquired through autoclaving twice at 120 °C for 60 min with a 24 h interval [50]. Each plastic nursery pot (10 cm outer diameter, 9 cm height, 8 cm bottom diameter) was sown with 20 healthy maize seeds (*Zea Mays*) and filled with 580 g soil or a mixture of soil and biochar (1–3 mm grain size). Three replicates were set for pot experiments. Maize seeds were all sterilized with 70% ethanol for 2 min, 1.2% (v/v) sodium hypochlorite for 10 min and washed in sterile distilled water before the experiment.

Biochar was purchased from Baide Water Purification Materials Co., Ltd. and was washed until no alkaline chemicals dissolved. The basic properties of biochar were as follows: pH 8.55 ± 0.18, mean pore size 5.06 ± 0.08 nm, C content 50.33 ± 0.17%, and P content 0.07 ± 0.01%. For the groups with FB treatment, the mixed plant-growth-promoting bacterial consortium (mentioned in Chapter 4.2) was added to the soil at the dosage of 10^9^ cfu. For all pots, plants were irrigated every day. The experiment was carried out in a plant growth room (room temperature 25 °C, light for 16 h, and humidity for 70~80%) and lasted for 21 days.

### 4.4. Determination of Indicators

The germination rates of maize seeds and the plant heights were recorded after the experiment. Enzyme activities of leaves such as malondialdehyde (MDA), proline, and superoxide dismutase (SOD) were determined using ELISA kits (Jiangsu Jingmei Biotechnology Co., Ltd., Jiangsu, China). Soil properties were determined in accordance with national standards: soil pH run NY/T 1121.2-2006, soil-soluble salt run LY/T 1251-1999, soil total phosphorus run NY/T 88-1988, soil total nitrogen run LY/T 1228-2015, soil organic matter run NY/T 85-1988, available phosphorus run NY/T 1121.7-2014, and available nitrogen run LY/T 1228-2015. Soil moisture content was determined by oven-drying and soil porosity was measured indirectly by soil bulk density [51,52]. The microbial community structure was analyzed through 16S rDNA high-throughput sequencing by Novogene Bioinformatic Technology Co. Ltd., Beijing, China.

### 4.5. Statistical Analysis

One-way ANOVA with the Duncan multiple-range test was employed to distinguish significant differences between different treatments. A Pearson correlation coefficient was conducted to analyze the correlations between soil quality and plant growth. All statistics were conducted with IBM-SPSS 25.0 software. Figures were drawn using Origin 2019b.

## 5. Conclusions

The isolated indigenous FB had relatively strong salt–alkali resistance and were capable of increasing soil nutrients, while their relative abundances were decreased due to indigenous microbial competition. The introduction of biochar could promote FB colonization and improved soil moisture and soil nutrient content effectively. Biochar combined with FB improved plant growth by lifting the nutrient levels in soil, optimizing the rhizospheric microbial community, increasing proline and SOD contents, and alleviating plant damage from high salinity. Among the treatments, 10% biochar and FB treatment had the greatest beneficial effects on soil properties and plant growth. The combined application of biochar and FB could be an effective and sustainable method to improve crop growth performance in saline–alkali soil.

## Figures and Tables

**Table 1:** Effects of single FB addition or the co-addition of biochar and FB on soil properties and fertility.

Treatment	Porosity (%)	Moisture (%)	pH	Soluble Salt (g/kg)	TP (g/kg)	TN (g/kg)	SOM (g/kg)	AP (mg/kg)	AN (mg/kg)
SP	34.31 ± 0.51 c	13.94 ± 0.38 c	8.69 ± 0.01 bc	3.09 ± 0.11 a	0.64 ± 0.04 c	0.17 ± 0.02 d	3.58 ± 0.20 cd	1.71 ± 0.11 c	13.49 ± 1.07 d
SSPB	34.07 ± 0.53 c	13.87 ± 0.40 c	8.65 ± 0.08 c	3.16 ± 0.11 a	0.68 ± 0.03 bc	0.22 ± 0.01 bc	2.82 ± 0.16 e	1.41 ± 0.00 cd	17.72 ± 1.45 c
SPB	34.21 ± 0.67 c	14.01 ± 0.50 c	8.69 ± 0.03 bc	3.03 ± 0.32 ab	0.70 ± 0.04 bc	0.19 ± 0.00 cd	3.06 ± 0.14 de	1.00 ± 0.20 d	14.56 ± 1.87 cd
SPBC1	34.63 ± 0.76 bc	14.62 ± 0.43 bc	8.58 ± 0.06 c	3.22 ± 0.29 a	0.69 ± 0.03 bc	0.24 ± 0.00 b	4.11 ± 0.30 c	1.68 ± 0.11 c	21.83 ± 1.96 b
SPBC5	36.42 ± 0.57 b	16.37 ± 0.36 a	8.80 ± 0.03 ab	2.78 ± 0.14 ab	0.75 ± 0.02 ab	0.25 ± 0.02 b	5.42 ± 0.28 b	2.26 ± 0.12 b	24.93 ± 2.02 ab
SPBC10	41.24 ± 0.84 a	15.88 ± 0.61 ab	8.92 ± 0.08 a	2.56 ± 0.13 b	0.81 ± 0.04 a	0.30 ± 0.03 a	7.58 ± 0.26 a	2.99 ± 0.32 a	28.94 ± 1.34 a

Notes: TP (total phosphorus); TN (total nitrogen); SOM (soil organic matter); AP (available phosphorus); AN (available nitrogen). Different lowercase letters show statistical differences among groups (*p* < 0.05). “S” represents “unsterilized soil”, “SS” represents “sterilized soil”, “P” represents “plant”, “B” represents “functional bacteria”, “C” represents “biochar”, and the number following the abbreviation represents the proportion of biochar mass to soil mass.

**Table 2:** The relative abundance of the added FB at genus level.

Treatment	*Bacillus* (%)	*Paenibacillus* (%)	*Azospirillum* (%)	*Rhizobium* (%)
S	0.223	0.028	0.135	1.258
SP	0.237	0.025	0.165	1.519
SSPB	0.473	0.060	0.241	1.982
SPB	0.301	0.023	0.208	1.917
SPBC1	0.306	0.020	0.231	4.681
SPBC5	0.446	0.010	0.261	4.581
SPBC10	0.391	0.030	0.544	4.846

Notes: “S” represents “unsterilized soil”, “SS” represents “sterilized soil”, “P” represents “plant”, “B” represents “functional bacteria”, “C” represents “biochar”, and the number following the abbreviation represents the proportion of biochar mass to soil mass.

**Table 3:** Pot experimental design for different treatments.

Soil	Plant	Bacteria	Biochar	Name	Purpose
Unsterilized	√			SP	Control
Sterilized	√	√		SSPB	To explore the function of FB without the interference of indigenous bacteria
Unsterilized	√	√		SPB	To explore the function of FB in soil with indigenous bacteria
Unsterilized	√	√	1%	SPBC1	To explore the combination effects of 1% biochar and FB
Unsterilized	√	√	5%	SPBC5	To explore the combination effects of 5% biochar and FB
Unsterilized	√	√	10%	SPBC10	To explore the combination effects of 10% biochar and FB

Notes: “S” represents “unsterilized soil”, “SS” represents “sterilized soil”, “P” represents “plant”, “B” represents “functional bacteria”, “C” represents “biochar”, and the number following the abbreviation represents the proportion of biochar mass to soil mass.

## Data Availability

All the data generated or analyzed during this study are included in this published article.

## Supplementary Materials

The following supporting information can be downloaded at: https://www.mdpi.com/article/10.3390/plants12020284/s1. Figure S1: Colony morphology (a,b), SEM photograph (c,d) and phylogenetic tree (e,f) of FN2 and DP3; Figure S2: The growth curves of (a) Bacillus megaterium and (b) *Azospirillum brasilense* under various salinity and alkalinity stresses; Table S1: Identification of physiological and biochemical characteristics of FN2 and DP3; Table S2: Carbon source utilization by FN2 and DP3; Table S3: Alpha diversity of microbial community composition.
