# Supplementary material for: Combination of Biochar and Functional Bacteria Drives the Ecological Improvement of Saline–Alkali Soil
Source: Plants (Basel). 2023 Jan 7;12(2):284. doi: 10.3390/plants12020284 (PMC9864812; doi:10.3390/plants12020284)
Supplement: Supplementary file 1 [file plants-12-00284-s001.zip › plants-2144080-supplementary.pdf]

Supplementary Information

Content:

Fig. S1 Colony morphology (a, b), SEM photograph (c, d) and phylogenetic tree (e, f) of FN2 and DP3. 2

Fig. S2 The growth curves of (a) *Bacillus megaterium* and (b) *Azospirillum brasilense* under various salinity and alkalinity stress. 7

Table S1 Identification of physiological and biochemical characteristics of FN2 and DP3. 3

Table S2 Carbon source utilization by FN2 and DP3. 4

Table S3 Alpha diversity of microbial community composition. 8

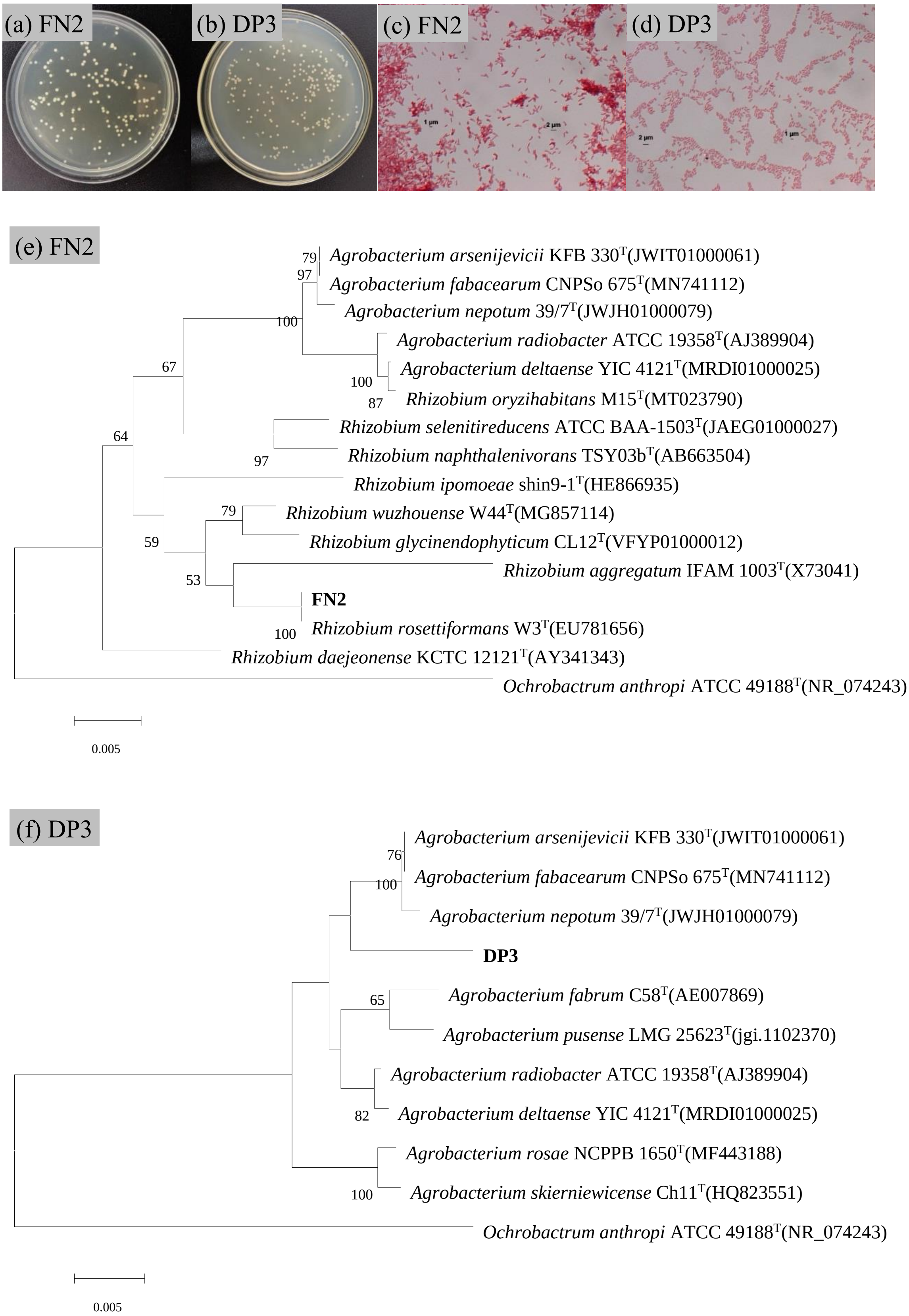

Fig. S1 Colony morphology (a, b), SEM photograph (c, d) and phylogenetic tree (e, f) of FN2 and DP3

Table S1 Identification of physiological and biochemical characteristics of FN2 and DP3.

Test	Composants Actifs	FN2	DP3
NO3	reduction desNitrates en nitrites	-	+
	reduction desNitrates en azote	-	-
TRP	L-tryptophane	-	-
GLU	D-glucose	-	-
ADH	L-arginine	-	-
URE	UREase	-	+
ESC	esculine citrate de fer	+	+
GEL	GELatine	-	-
PNPG	4-nitrophenyl-β-D-galactopyranoside	+	+
GLU	D-glucose	+	+
ARA	L-arabinose	+	+
MNE	D-mannose	+	+
MAN	D-mannitol	+	+
NAG	N-acetyl-glucosamine	+	+
MAL	D-maltose	+	+
GNT	potassium gluconate	-	+
CAP	acide caprique	-	-
ADI	acide adipique	-	-
MLT	acide malique	-	+
CIT	trisodium citrate	+	-
PAC	acide phenylacetique	-	-

Note: +: positive; -: negative; w: weakly positive.

Table S2 Carbon source utilization by FN2 and DP3

Hole number	Types of carbon sources	FN2	DP3
A1	Negative Control	-	-
A2	Dextrin	-	-
A3	D-Maltose	-	w
A4	D-Trehalose	+	w
A5	D-Cellobiose	-	+
A6	Gentiobiose	w	+
A7	Sucrose	+	w
A8	D-Turanose	w	w
A9	Stachyose	w	w
A10	Positive Control	+	+
A11	pH 6	+	w
A12	pH 5	-	-
B1	D-Raffinose	+	w
B2	α-D-Lactose	-	w
B3	D-Melibiose	w	w
B4	β-Methyl-DGlucoside	w	+
B5	D-Salicin	w	w
B6	N-Acetyl-DGlucosamine	+	+
B7	N-Acetyl-β-DMannosamine	+	+
B8	N-Acetyl-DGalactosamine	+	-
B9	N-Acetyl Neuraminic Acid	-	-
B10	1% NaCl	+	+
B11	4% NaCl	+	w
B12	8% NaCl	-	-
C1	α-D-Glucose	-	w
C2	D-Mannose	-	w
C3	D-Fructose	w	+
C4	D-Galactose	w	+
C5	3-Methyl Glucose	-	-
C6	D-Fucose	w	+
C7	L-Fucose	w	+
C8	L-Rhamnose	w	+

C9	Inosine	w	w
C10	1% Sodium Lactate	-	+
C11	Fusidic Acid	-	w
C12	D-Serine	-	-
D1	D-Sorbitol	+	w
D2	D-Mannitol	w	w
D3	D-Arabitol	w	w
D4	myo-Inositol	+	+
D5	Glycerol	+	-
D6	D-Glucose-6-PO4	-	+
D7	D-Fructose-6-PO4	-	+
D8	D-Aspartic Acid	-	-
D9	D-Serine	-	-
D10	Troleandomycin	w	+
D11	Rifamycin SV	+	+
D12	Minocycline	-	-
E1	Gelatin	-	-
E2	Glycyl-L-Proline	w	w
E3	L-Alanine	+	+
E4	L-Arginine	+	w
E5	L-Aspartic Acid	+	+
E6	L-Glutamic Acid	+	+
E7	L-Histidine	+	w
E8	L-Pyroglutamic Acid	+	+
E9	L-Serine	w	+
E10	Lincomycin	w	+
E11	Guanidine HCl	w	w
E12	Niaproof 4	-	-
F1	Pectin	w	-
F2	D-Galacturonic Acid	w	+
F3	L-Galactonic Acid Lactone	-	+
F4	D-Gluconic Acid	w	+
F5	D-Glucuronic Acid	w	+
F6	Glucuronamide	w	w

F7	Mucic Acid	-	-
F8	Quinic Acid	+	-
F9	D-Saccharic Acid	-	w
F10	Vancomycin	-	w
F11	Tetrazolium Violet	w	+
F12	Tetrazolium Blue	+	+
G1	p-Hydroxy-Phenylacetic Acid	-	-
G2	Methyl Pyruvate	w	w
G3	D-Lactic Acid Methyl Ester	-	-
G4	L-Lactic Acid	+	+
G5	Citric Acid	+	-
G6	α-Keto-Glutaric Acid	w	-
G7	D-Malic Acid	+	+
G8	L-Malic Acid	+	+
G9	Bromo-Succinic Acid	+	-
G10	Nalidixic Acid	+	+
G11	Lithium Chloride	+	+
G12	Potassium Tellurite	+	+
H1	Tween 40	w	-
H2	γ-Amino-Butryric Acid	+	w
H3	α-Hydroxy-Butyric Acid	w	w
H4	β-Hydroxy-D,L-butyric Acid	+	-
H5	α-Keto-Butyric Acid	-	w
H6	Acetoacetic Acid	w	w
H7	Propionic Acid	+	w
H8	Acetic Acid	+	+
H9	Formic Acid	+	+
H10	Aztreonam	+	+
H11	Sodium Butyrate	+	+
H12	Sodium Bromate	-	-

Note: +: positive; -: negative; w: weakly positive.

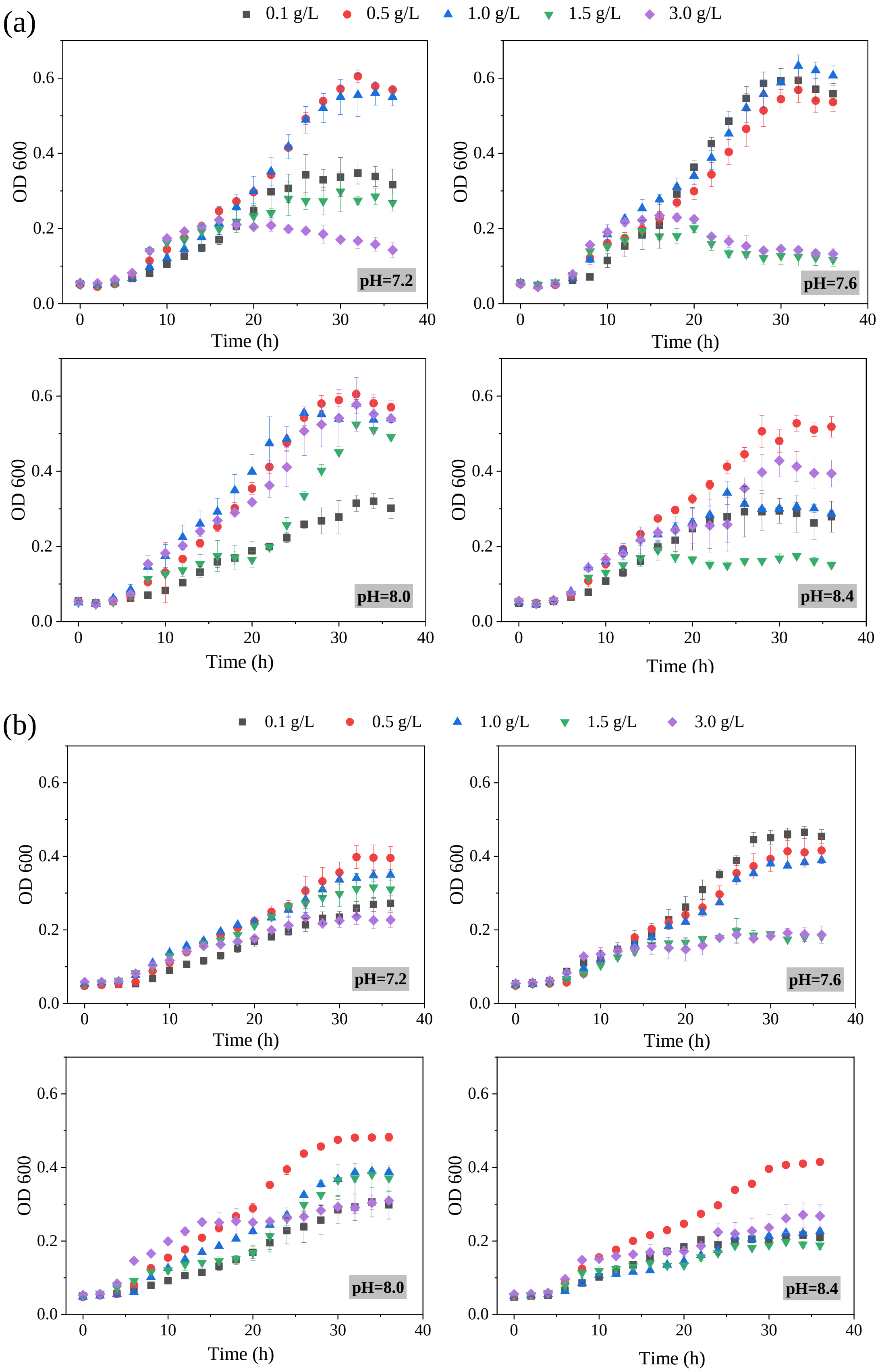

Fig. S2 The growth curves of (a) *Bacillus megaterium* and (b) *Azospirillum brasilense* under various salinity and alkalinity stress

Table S3 Alpha diversity of microbial community composition

Treatment	OTUs	Chao1	ACE	Shannon	Simpson
S	2099	2274.029	2289.660	8.295	0.987
SP	1356	1472.179	1497.928	6.727	0.957
SSPB	1079	1245.870	1352.311	5.917	0.946
SPB	1461	1618.093	1729.523	6.705	0.946
SPBC1	1038	1215.100	1235.745	6.157	0.951
SPBC5	1062	1215.720	1261.121	5.893	0.922
SPBC10	971	1171.536	1218.934	6.081	0.959
